# Peritoneal Bridging Versus Nonclosure in Laparoscopic Ventral Hernia Repair: A Randomized Controlled Trial

**DOI:** 10.1097/AS9.0000000000000257

**Published:** 2023-02-02

**Authors:** Fathalla Ali, Gabriel Sandblom, Bianka Forgo, Göran Wallin

**Affiliations:** From the * Faculty of Medicine and Health, Department of Surgery, Örebro University, Örebro, Sweden; † Department of Surgery, Karlskoga Hospital, Karlskoga, Sweden; ‡ Department of Clinical Science and Education Södersjukhuset, Karolinska Institutet, Stockholm, Sweden; § Department of Surgery, Southern Hospital (Södersjukhuset), Stockholm, Sweden; ‖ Department of Radiology, Örebro University Hospital, Faculty of Medicine and Health, Örebro University, Örebro, Sweden.

**Keywords:** epigastric hernia, incisional hernia, IPOM with fascia closure, IPOM with peritoneal bridging, laparoscopic hernia repair, simple IPOM, umbilical hernia, ventral hernia

## Abstract

**Introduction:**

Postoperative seroma and pain are common problems following laparoscopic intraperitoneal onlay mesh (IPOM) repair of ventral hernias. These adverse outcomes may be avoided by dissecting and using the peritoneum in the hernial sac to bridge the hernia defect.

**Methods:**

This was a patient- and outcome assessor-blinded, parallel-design, randomized controlled trial comparing nonclosure and peritoneal bridging approaches in patients scheduled for elective midline ventral hernia repair. The primary endpoint was seroma volume on ultrasonography. The secondary endpoints were postoperative pain, recurrence, and complications.

**Results:**

Between November 2018 and December 2020, 112 patients were randomized, of whom 60 were in the nonclosure group and 52 were in the peritoneal bridging group. The seroma volume in the nonclosure and peritoneal bridging groups were 17 cm^3^ (6–53 cm^3^) versus 0 cm^3^ (0–26 cm^3^) at 1-month follow-up (*P* = 0.013). The median volume was zero at the 3-, 6-, and 12-month follow-ups in both groups. No significant differences were observed in early postoperative pain (*P* = 0.447) and in recurrence rate (*P* = 0.684). There were 4 (7%) and 1 (2%) perioperative complications that lead to reoperations in simple IPOM (sIPOM) and IPOM with peritoneal bridging (IPOM-pb), respectively.

**Conclusions:**

Seroma was less prevalent after IPOM-pb at 1-month follow-up compared with sIPOM, with similar postoperative pain 1 week after index surgery in both groups. At subsequent follow-ups, the differences in seroma were not statistically significant. Further studies are required to confirm these results. Trial registration (NCT04229940).

## INTRODUCTION

Laparoscopic ventral hernia repair (LVHR) has gained acceptance since its introduction by LeBlanc^1^ in 1993. This is mainly due to the favorable outcomes of seroma, pain/discomfort, aesthetics, surgical site infections, and recurrences compared with open ventral hernia repair.^2–4^

However, there are considerable controversies regarding the different approaches to LVHR. A commonly used laparoscopic approach is simple intraperitoneal onlay mesh (sIPOM) repair, in which an intraperitoneal onlay mesh (IPOM) is placed with at least 5 cm overlapping the hernia defect (without defect closure). Nevertheless, this approach creates space between the mesh and the overlying hernial sac, which is believed to be the cause of seroma formation (Fig. 2B).^5^

**FIGURE 1.**
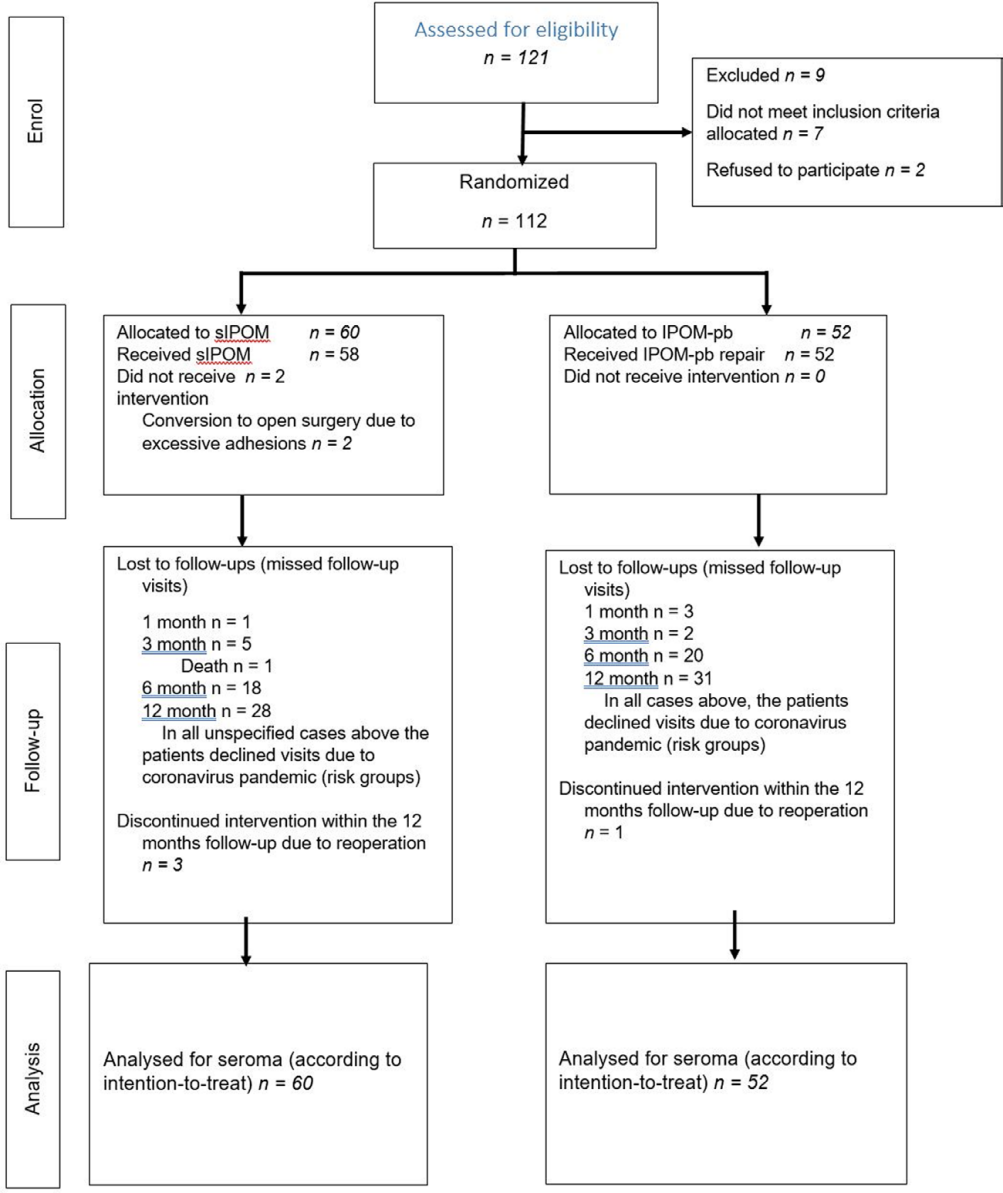
Flow chart.

Most patients develop some elements of seroma after laparoscopic hernia repair because of the potential space created within the hernia sac. The incidence of seroma detected using ultrasonography in the early postoperative period has been reported to be close to 100%.^6,7^ While nearly all patients may develop seroma, most postoperative seroma formations have been reported to resolve spontaneously without any intervention.^6^ Clinically significant seromas that require intervention have been reported to be in 3%–17% of cases after LVHR.^5^

Some seromas may become clinically relevant if it results in patient dissatisfaction, discomfort, pain, and/or surgical site infection, which may lead to subsequent contamination and removal of prosthesis.^8–10^

**FIGURE 2.**
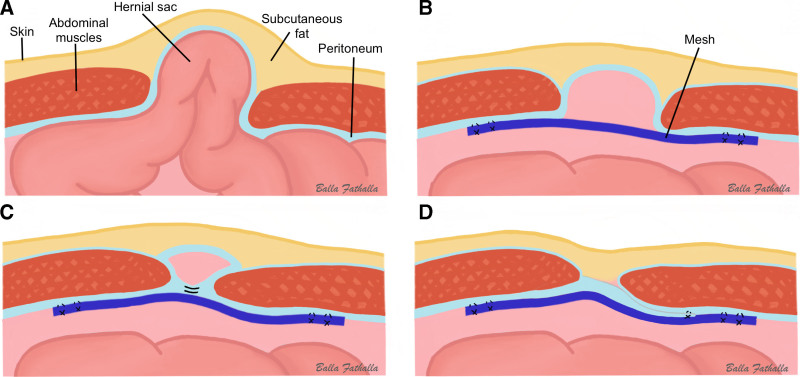
Schematic overview of the 3 difference types of IPOM hernia repair approaches. A, Hernial sac with bowel content without any repair. B, Final result of nonclosure approach (sIPOM). C, End result of IPOM with primary fascial closure (IPOM-plus). D, Final result of IPOM with peritoneal bridging approach (ie, peritoneum in hernial sac dissected and bridged over the hernia defect) (IPOM-pb).

**FIGURE 3.**
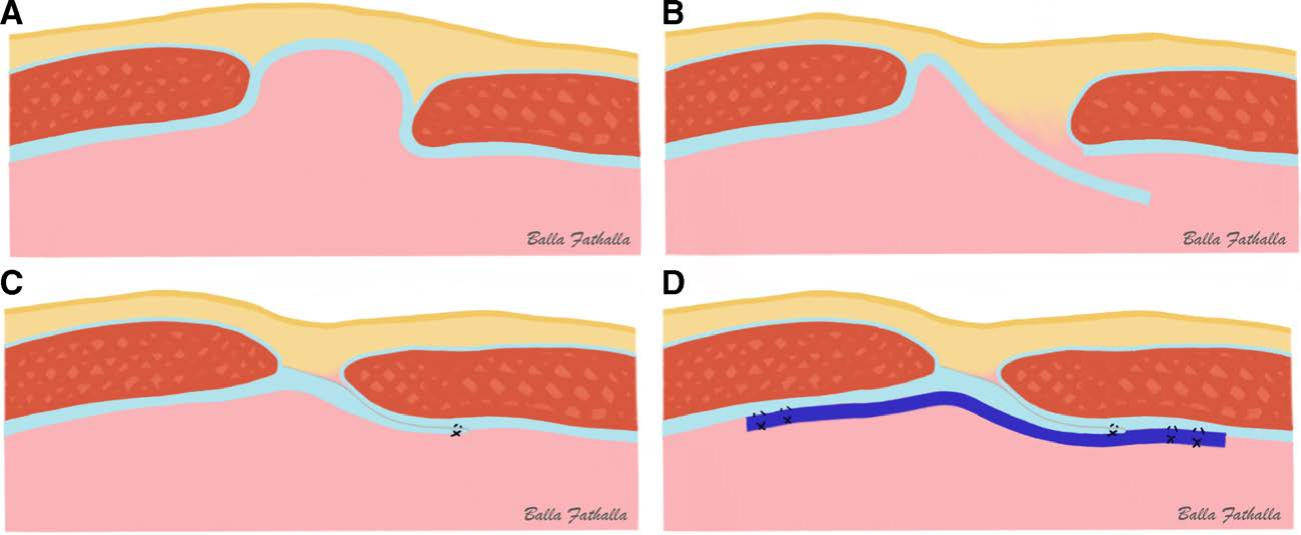
Stages of IPOM with peritoneal bridging approach. A, Hernia with bowel content reduced, and no hernia repair performed. B, The peritoneum of hernial sac is dissected halfway through the hernial sac and pulled down to bridge the hernial defect. C, The dissected peritoneum is pulled down to bridge the hernial defect and is sutured to overlap the hernial defect. D, A mesh is applied such that it is overlapping the hernial defect by minimum of 5 cm with a double crown technique.

An alternative for defect closure with the IPOM technique is to dissect the peritoneum halfway through the hernial sac, which is then bridged across the hernial defect and sutured to the abdominal wall (IPOM-pb) (Fig. 2D and Fig. 3B–D). This results in closure of the defect and the additional benefit of further reducing the dead space within the hernial sac without causing tension from approximating the defect margin using sutures, as in IPOM-plus. Hence, hypothetically, with the IPOM-pb approach, favorable outcomes of a lower risk of recurrence of IPOM-plus, less pain/discomfort of sIPOM, and further reduction of seroma would be achieved.

A method description with schematics and a comparison of these 3 IPOM techniques were described in detail in a previous study.^11^

This randomized controlled trial aimed to compare the risk of seroma formation and other postoperative outcomes between IPOM with nonclosure (sIPOM) and IPOM with peritoneal bridging (IPOM-pb). As the main purpose of the peritoneal bridging was to reduce the dead space, postoperative seroma was considered primary endpoint.

## METHODS

This study was conducted as a single-center randomized controlled trial. Patients scheduled for elective laparoscopic hernia repair for abdominal ventral hernia at Karlskoga Hospital, Hernia Center, were consecutively screened for inclusion and invited to participate (Fig. 1).

The inclusion criteria were patients ≥18 years old scheduled for planned laparoscopic hernia repair for midline ventral hernia. Midline ventral hernia was defined as any hernia in the anterior abdominal wall between the xiphoid process and superior pubic ramus. Exclusion criteria were inability to provide informed consent, emergency surgery (for irreducible, strangulated, or obstructed hernia), serious comorbidities, defect size of smaller than 3 cm or larger than 10 cm in diameter, preoperative suspicion of extensive adhesions, pregnancy or intended pregnancy, and body mass index (BMI) exceeding 40 kg/m^2^.

The study was approved by the Ethics Review Board of Uppsala University (2018/478) and all patients provided written informed consent for more than 24 hours before surgery. This trial was registered at ClinicalTrials.gov (NCT04229940). The Consolidated Standards of Reporting Trials guidelines^12^ were followed.

### Trial Randomization and Blinding

The patients were randomized into 2 IPOM alternatives: sIPOM without defect closure and IPOM-pb. Eligible patients were randomized using a simple randomization method. No blocking was done. The patients were enrolled by the surgeon planning the repair according to the protocol, and the random assignment of participants according to the sequence allocation was completed by the secretary of the Department of Surgery. The random allocation was concealed using sequentially numbered opaque sealed envelopes. The sonographer was blinded to the surgical technique used.

### Surgical Procedure and Intervention

All laparoscopic hernia repair procedures were performed by a single, experienced surgeon. The abdominal cavity was insufflated to 12 mm Hg using a Veress needle; one 12-mm port and two 5-mm ports were used. Adhesiolysis was performed as required. The fascial defect was measured using disposable sterile tape at an intra-abdominal pressure of 8 mm Hg.

The hernia defect was repaired according to the following allocation: sIPOM without defect closure or IPOM-pb. In the sIPOM technique, an IPOM was placed with a 5 cm overlap of the hernia defect margin on all sides without any closure of the defect prior to mesh placement (Fig. 2B). In the IPOM-pb technique, the defect was closed, and peritoneal coverage of the dead space within the hernial sac was eliminated by peritoneal bridging prior to mesh placement (Fig. 2D). In contrast to sIPOM, no communication was left between the abdominal cavity and hernia sac. Peritoneal bridging was achieved by dissecting the peritoneum halfway through the peritoneal sac (Fig. 3B), which was subsequently brought down together with the sac (Fig. 3B, C), bridged across the defect, and sutured (Fig. 3D). Both sIPOM and IPOM-pb procedures are described step by step in a previously published article.^11^

Patients whose surgery was converted to open surgery were included in the study and analyzed according to the intention-to-treat principle.

Local anesthetic (20 mL ropivacaine 7.5 mg/mL) infiltration was administered routinely. The skin was closed with intradermal 4.0 Monocryl (Ethicon, Cornelia, GA) sutures. All patients were instructed to wear an abdominal elastic binder continuously for 14 days after the index surgery and in the subsequent 14 days only during the daytime.

### Patient Assessments

The patients included in the study underwent ultrasound examination of the abdominal wall at 1, 3, 6, and 12 months postoperatively. All examinations were performed by the same observer (B.F., radiology trainee with 3 years of experience) and equipment (Siemens Acuson 3000). The sonographer was blinded to the surgical technique used. None of the patients were prepared for ultrasound examination. The patients were examined in the supine position. Seromas with hyperechogenic content were also evaluated with the patient in the left lateral decubitus position to differentiate moving particles from the solid content fixed in the seroma.

B-mode ultrasound was performed on the entire ventral abdominal wall using a high-frequency, 5.5–18 MHz linear array transducer (Siemens Acuson 18L6 HD) in all patients. A 4–9 MHz linear array transducer (Siemens Acuson 9L4) was used in obese patients where the 5.5–18 MHz linear probe failed to visualize the mesh. All pathologies anterior to the mesh were evaluated by using linear transducers. A 1.5–6 MHz convex transducer (Siemens Acuson 6C1 HD) was used to detect seromas posterior to the mesh and, if necessary, to measure the size of large seromas. For each ultrasound control, the presence or absence of seroma was noted. A seroma was defined as a delimited fluid collection located in the abdominal wall with dimensions >1 cm in the transverse, coronal, and sagittal planes.

The width (W), length (L), and depth (D) of fluid collection were measured, and the dimensions were multiplied to calculate the volume (V) of the seroma according to the following formula^13,14^:


$$\begin{array}{l} V=W\times L\times D\;x\;\pi /6 \end{array}$$

In cases in which several seromas were detected, the volume of each seroma was calculated separately using the aforementioned formula. The total seroma volume in these cases was calculated by adding individual fluid collection volumes.

### Outcomes

The primary endpoint was seroma volume detected on ultrasound at 1-month follow-up. The secondary outcomes included seroma detection using ultrasonography at 3, 6, and 12 months of follow-up. Other secondary outcomes were intraoperative data, such as operative time, conversion, intraoperative and postoperative complications, recurrence, and postoperative pain.

### Statistical Analysis

Data were analyzed according to the intention-to-treat principle, and nonparametric statistics were applied because the data were not normally distributed (evaluated using the Kolmogorov-Smirnov test). Categorical variables (eg, presence of seroma) were compared using the χ^2^ test or Fisher exact test and are presented as counts (percentages). Continuous variables (eg, seroma volume) were compared using the Mann-Whitney *U* test and presented as median (interquartile range) or mean (SD). The significance level was set at *P* < 0.05.

This study is based on the assumption of superiority. The sample size calculation was based on 80% power (1–β) and a significance level of 0.05 (2-sided α). Assuming that the seroma volume was 240 mL in the peritoneal bridging group, 300 mL in the sIPOM group, and a SD of 100 mL in both groups, a total of 88 patients would be needed. To compensate for this dropout rate, 115 patients were included in the study.

Statistical analyses were performed using SPSS (Statistical Package for Social Sciences for Windows, version 28.0; IBM Corp., Armonk, NY).

## RESULTS

Patients scheduled for elective laparoscopic hernia repair were consecutively recruited between November 2018 and December 2020 at the Karlskoga Hospital. A total of 112 patients were consecutively randomized, of which 60 and 52 were assigned to the sIPOM and IPOM-pb groups, respectively. Two patients in the sIPOM group did not receive the intended laparoscopic sIPOM but underwent conversion to open surgery due to excessive adhesions. Additionally, 5 patients underwent reoperations: 3 in the sIPOM group and 1 in the IPOM-pb group. All patients were analyzed according to the intention to treat. The reasons for conversion and/or reoperation are listed in Table 1.

**TABLE 1.** Overview and Reasons for Conversions and/or Reoperations

Method	Conversion	Reason for Conversion	Reoperation	Reason for Reoperation	Post-Reoperation Follow-Up	Death
Planned sIPOM	Yes	Extensive adhesion	No			
		Small bowel ischemia				
sIPOM	No		Yes	Bleeding from omentum	Bleeding source was stopped with diathermy	
Planned sIPOM	Yes	Extensive adhesions	Yes	Intestinal Injury	Multiple reoperation and VAC therapy	At 3 months
					Long hospital stay	
IPOM-pb	No		Yes	Intestinal injury	Long hospital stay	
sIPOM	No		Yes	Ileus		
sIPOM	No		Yes	Ileus	Infected seroma	
					Long hospital stay	

The patients in the third and fourth row were operated at Orebro University Hospital.

VAC indicates vacuum-assisted closure.

There were 2 conversions to open surgery due to excessive adhesions, during which planned sIPOM was not performed prior (Table 1).

One patient who underwent conversion and 4 other patients who received the intended randomized procedure underwent reoperation after the respective index surgery. The patient scheduled for sIPOM who underwent conversion deteriorated postoperatively because of intestinal injury, followed by multiple laparotomies and vacuum-assisted closure therapy. The patient died 3 months after the index surgery. Of the 3 completed sIPOM patients, 1 underwent reoperation due to postoperative pain and suspected bleeding, in which intraoperatively detected bleeding in the omentum was found and electrocauterized. The other 2 patients who underwent the sIPOM procedure underwent reoperations because of intestinal obstruction. In the IPOM-pb group, reoperation was performed at Örebro University Hospital after the index IPOM-pb was completed because of intestinal injury, which was followed by 2 months of hospital stay.

Patient demographics are summarized in Table 2. On average, patients in the IPOM-pb group were 3 years older than those in the sIPOM group.

**TABLE 2.** Baseline Characteristics

	sIPOM (N = 60)	IPOM-pb (N = 52)	All Repairs (N = 112)
Gender, n (%)			
Male	35 (58)	26 (50)	61 (54)
Female	25 (42)	26 (50)	51 (46)
Age, years, median (IQR)	59 (50–69)	62 (48–73)	60 (48–72)
BMI, kg/m^2^, mean (SD)	30.1 (3.9)	29.6 (5.3)	29.8 (4.6)
COPD*, n (%)	1 (2)	1 (2)	2 (2)
Smoking*, n (%)	4/44	6/36	10/80
Diabetes*, n (%)	3 (5)	5 (10)	8 (7)
ASA score*, n (%)			
ASA-I	29 (48)	21 (40)	50 (45)
ASA-II	27 (45)	22 (42)	49 (44)
ASA-III	4 (7)	9 (17)	13 (12)
Type of hernia, n (%)			
Epigastric	9 (15)	6 (12)	15 (13)
Umbilical	14 (23)	10 (19)	24 (21)
Incisional	37 (62)	36 (69)	73 (65)

*Data for COPD, smoking, diabetes, and ASA score were obtained from the local medical records.

ASA indicates American Society of Anesthesiologists; COPD, chronic obstructive pulmonary disease; IQR, interquartile range.

The operative data are summarized in Table 3. The median operative time was 20 min longer in the IPOM-pb group than in the sIPOM group (*P* = 0.692) because of additional steps prior to mesh placement. The median defect diameter was 1 cm smaller in the sIPOM group than in the IPOM-pb group (*P* = 0.065).

**TABLE 3.** Perioperative Outcomes

	sIPOM (N = 60)	IPOM-pb (N = 52)	All Repairs (N = 112)	*P*
Operative data, median (IQR)				
Operative time, minutes	47 (39–61)	67 (51–72)	55 (44–72)	
Defect diameter, cm	5.0 (4.5–6.0)	6.0 (5.0–7.0)	5.5 (5.0–7.0)	
Pain, 1–10 VAS scale†, median (IQR)				
At hospital discharge	2 (1–2)	2 (1–2)	2 (1–2)	
1 week‡	1 (1–1)	1 (1–2)	1 (1–2)	
Emergency department visit, n (%)				
POD 1–30§	11 (18)	4 (8)	15 (13)	
Symptomatic seroma aspirated to decrease symptoms	1 (2)	0 (0)	1 (1)	
Infected seroma	2 (3)	0 (0)	2 (2)	
Surgical site infection	1 (2)	0 (0)	1 (1)	
Thrombophlebitis	1 (2)	0 (0)	1 (1)	
Pain/discomfort	5 (8)	2 (4)	7 (6)	0.447
Unrelated causes	1 (2)	2 (4)	3 (3)	
Recurrence, n (%)	4 (7)	2 (4)	6 (5)	0.684
CT-verified	1 (2)	1 (2)	2 (2)	
Clinical or by ultrasound	3 (5)	1 (2)	4 (4)	
Deaths‖, n (%)	1 (1)	2 (4)	3 (3)	
Sonographic follow-up visits				
Incidence of seroma, count (%)				
1st month	52/56 (93)	30/48 (63)	82/104 (79)	< 0.001
3rd month	19/52 (37)	22/49 (45)	41/101 (41)	0.393
6th month	10/39 (26)	10/31 (32)	20/70 (29)	0.543
12th month	3/29 (10)	5/20 (25)	8/49 (16)	0.245
Median seroma volume, cm^3^ (IQR)				
1st month¶	17 (6–53)	0.0 (0–26)	6.0 (0–43)	0.013
3rd month	0 (0–1)	0 (0–1)	0 (0–1)	0.504
6th month	0 (0–0)	0 (0–0)	0 (0–0)	0.625
12th month	0 (0–0)	0 (0–0)	0 (0–0)	0.169

*The reasons the respective conversions and reoperations in each group are listed in Table 1.

†VAS (1–10).

‡Postoperative pain was followed up by phone 1 week after index surgery.

§POD period.

‖A total of 3 patients died. The cause of deaths in the IPOM-pb group was unrelated to, and in both cases occurred 2 years after, index surgery. In the sIPOM group, the patient planned for sIPOM had extensive adhesions that necessitated conversion to open surgery. The patient deteriorated postoperatively, suspected to have intestinal injury, multiple laparotomies and VAC therapy because of intestinal injury. The patient died 3 months after index.

¶There were 2 outliers in total at 1-month follow-up, both in the IPOM-pb group. One patient had 969 cm^3^ and another patient 454 cm^3^ of seroma. *P* value for seroma volume was obtained with Mann-Whitney *U* test.

CT indicates computer tomography; IQR interquartile range; POD, postoperative day; VAS, Visual Analog Scale.

The postoperative data are summarized in Table 3. The median seroma volume measured by ultrasound at 1-month follow-up was significantly greater in the sIPOM group than in the IPOM-pb group (*P* = 0.013).

There were 2 outliers in total at 1-month follow-up, both in the IPOM-pb group. One patient had a volume of 969 cm^3^ and another patient 454 cm^3^ of seroma. The seroma in both patients was substantially reduced at 3 months follow-up: in the former patient to 30 cm^3^, and in the latter patient, it resolved completely.

Patient-reported postoperative pain was similar in both groups on the same day as hospital discharge in the sIPOM and IPOM-pb groups (median Visual Analog Scale [VAS] 2 vs 2, respectively). Likewise, postoperative pain 1 week after surgery was also similar (median VAS 1 vs 1). However, 20% of patients in total did not report the pain the same day as hospital discharge, and there were missing data for approximately 25% of patients for the 1-week postoperative pain follow-up.

Postoperative seroma detected by ultrasound at 1-month follow-up was proportionally more prevalent in sIPOM group compared with IPOM-pb (93 % vs 63%; *P* < 0.001).

**FIGURE 4.**
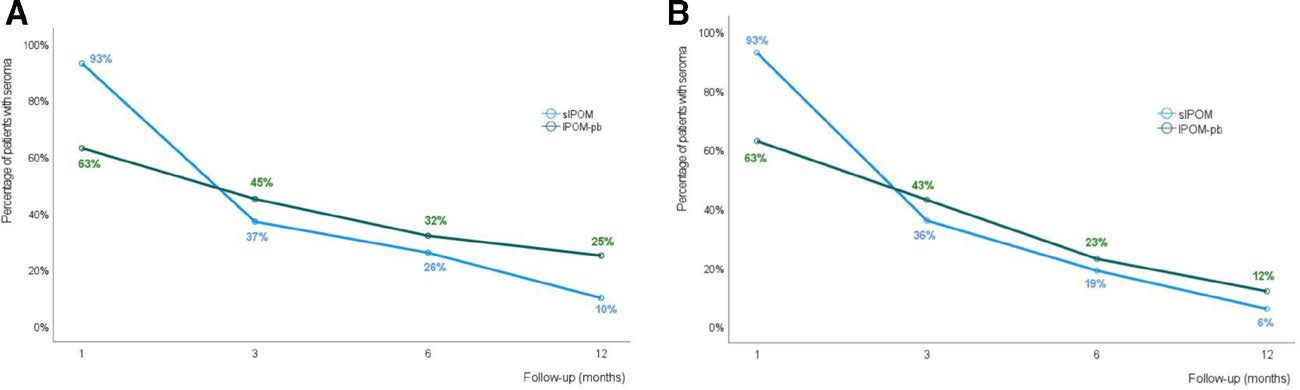
Seroma prevalence at follow-up. A, Percentage of patients with seroma without adjusting for lost to follow-ups in respective IPOM group. B, Percentage of patients with seroma after adjusting for the lost to follow-ups in respective IPOM group.

At subsequent visits, seroma was more prevalent in the IPOM-pb group than in the sIPOM group (Fig. 4A). At 3 months follow-up, seroma were 19 of 52 (37%) versus 22 of 49 (45%) in sIPOM and IPOM-pb, respectively (*P* = 0.393). Likewise, there were more patients with seroma in the IPOM-pb group at 6 months follow-up (10/39, 26% vs 10/31, 32%; *P* = 0.543) and 12 months follow-up (3/29, 10% vs 5/20, 25%; *P* = 0.245).

The percentage of patients with seroma in each group over time is shown in Figure 4A. Most of these patients had no seroma detected on ultrasound during the previous follow-up period (Table 3 in the Supplementary Results Material, http://links.lww.com/AOSO/A207). There were no cases of seroma development after the absence of a postoperative seroma at an earlier follow-up visit. It is generally expected that most postoperative seromas occur in the early postoperative period and spontaneously resolve over time.^6,7,11,13,15^

Thus, it could be assumed that patients who missed later follow-up examinations and had no seroma detected by ultrasound in the earlier postoperative visit would also have no seroma detected by ultrasound at the subsequent missed follow-ups if they had come. Based on this assumption, the percentage of patients with seroma in the 2 graphs in Figure 4A would converge at later postoperative follow-ups (Fig. 4B) (Table 4 in Supplementary Results Material, http://links.lww.com/AOSO/A208). This suggests that when eliminating the effect of lost to follow-up, the reabsorption rate would be more similar in both groups during the last 6 months of the postoperative period.

## DISCUSSION

The present randomized controlled trial showed that the median seroma volume was higher in the sIPOM group than in the IPOM-pb group during the first month of follow-up (17.0 vs 0.0; *P* = 0.013). Nevertheless, the clinical relevance of seroma may still be debated because complicated seroma, in terms of symptomatic seroma that required aspiration or infected seroma, was found to be only 3% in sIPOM group only (3/60 vs 0/52; *P* < 0.247) and none in the IPOM-pb group. The median seroma volume was zero at the subsequent follow-up in both groups.

Although the prevalence of seroma at the 1-month follow-up was higher, the reabsorption of seroma 3 months after surgery was faster in sIPOM than in IPOM-pb. The prevalence of seroma decreased from 93% to 37% in sIPOM group and from 63% to 45% in IPOM-pb group 3 months after index surgery (Table 3). Although the cause is not understood, one possible reason could be that the open communication of the hernia sac from the open defect (ie, nonclosure of the hernia defect) in sIPOM allows dissipation of seroma formation into the abdomen, thereby facilitating faster reabsorption of the seroma.

Several methods have been suggested to reduce seroma formation,^5^ most of which aim to reduce the space created between the mesh and overlying hernia sac.^5,16^ These include electrical cauterization of the hernia sac, use of fibrin sealant, primary defect closure of the hernia defect (IPOM-plus), and peritoneal dissection halfway through the peritoneal sac, which is subsequently used to bridge the hernia defect (IPOM-pb).

The evidence for electrical cauterization to reduce seroma formation seems promising, but it is based on a few studies.^5^ Prassas and Schumacher^17^ reported rate of seroma to be 0% after electrical cauterization and 25% in control group, and Tsimoyiannis et al^18^ reported 1.9% after electrical cauterization and 25% in the control group. Both were retrospective studies with 174 patients in total, and the results of both studies were statistically significant. The rationale behind this is that the seroma is reduced by destroying the mesothelial cells (which produce inflammatory exudate) and the formation of adhesions to reduce the dead space between the mesh and the cauterized surface of the hernia sac.^17^ However, these results should be interpreted with caution as they are based on a few smaller studies, and larger multicentre studies are needed to confirm these results.^5^

Fibrin sealants have also been suggested to reduce seroma formation. In 1 prospective study, 1 mL fibrin sealant (Tissucol Duo) for every 16 cm^2^ prosthesis was injected percutaneously into the hernial sac. The rate of seroma found on computer tomography scans for the fibrin sealant group was 64% at 7 days and 12% at 3 months, and for the nonfibrin sealant group, 92% at 7 days and 24% at 3 months.^19^

Primary fascial closure (IPOM-plus) is another method used to reduce seroma formation that approximates fascial defect edges prior to mesh placement. By approximating the defect edges (ie, closure of the fascia defect), the space within the hernial sac was reduced (Fig. 2C). This method provides the additional benefit of a greater surface contact area between the mesh and the abdominal wall, which is thought to result in a lower recurrence rate and mesh migration.^9,16,20,21^ However, although IPOM-plus is thought to result in fewer seromas than sIPOM, previous studies show conflicting results.^5^ Additionally, IPOM-plus may result in more pain, discomfort, and/or fatigue in the early postoperative period because of the surgical tension created by the primary defect closure.^15,22,23^

To the best of our knowledge, there have been 2 previous studies on repeated sonographic seromas after LVHR. A previous study by Piazzese et al^13^ reported seroma incidence detected by ultrasound that was repeated in 1-, 3-, and 6-month periods and one by Susmallian et al^7^ with repeated ultrasound at 1 and 3 months follow-up (Table 1 in Supplementary Results Material, http://links.lww.com/AOSO/A205). There is no known previous study in which all seromas were assessed by ultrasound 12 months after index surgery. The incidence of seroma reported by Piazzese et al^13^ was lower than that reported by Susmallian et al^7^ and the results of the present study (Table 1 in Supplementary Results Material, http://links.lww.com/AOSO/A205).

It is somewhat unclear why there was a great discrepancy between the sonographic incidence in the present study and that in a previous study by Piazzese et al^13^ (Table 1 in Supplementary Results Material, http://links.lww.com/AOSO/A205). A previous study linked a higher BMI to a greater incidence of seroma^24^; thus, one possible contributing factor could be that the patients in this study had an average BMI of approximately 5 kg/m^2^ higher than the patients in the aforementioned study by Piazzese et al.^13^

In designing the study, postoperative pain was followed up on the day of discharge from the hospital and 1 week after the index surgery. This decision was based on the results of a previous study that showed the greatest difference in postoperative pain between the different IPOM approaches within the first 7 days after surgery.^25^ However, in hindsight, it would be interesting to study the postoperative pain and/or discomfort on a daily basis within the first 7 days after surgery between sIPOM and IPOM-pb to analyze if there was a significant difference in patient’s postoperative experience in the form of postoperative pain, discomfort, any physical limitations due to pain, and/or fatigue within the first week after surgery. We chose to include patients with defects up to 10 cm, which is in accordance with Society of American Gastrointestinal and Endoscopic Surgeons guidelines for laparoscopic ventral hernias.^25^ Nevertheless, hernias with defects close to 10 cm may require great efforts to repair with laparoscopic technique and other methods of closing the defect than peritoneal bridging should be considered. None of the patients included in the present study had a defect larger than 8 cm.

This study has some limitations. First, patient enrollment began at the beginning of the coronavirus pandemic and several patients decided not to attend the follow-up visits as the study went on, especially since several patients belonged to the risk groups. The number of patients lost to follow-up increased as the study went on: at 1-month follow-up, 2% versus 4% were lost to follow-up in the sIPOM and IPOM-pb groups, respectively, which increased up to approximately half of the patients at the 12-month follow-ups (49% for sIPOM and 61% for IPOM-pb). The number of patients lost to follow-up at respective months for each group is presented in Table 2 in the Supplementary Results Material (http://links.lww.com/AOSO/A206).

In conclusion, the results of this study showed a lower incidence of seroma with peritoneal bridging compared with the simple nonclosure technique 1 month after the index surgery. Nevertheless, future studies are required to compare the postoperative outcomes of IPOM with nonclosure and peritoneal bridging techniques more accurately, including a comparison of the potential benefits of lower seroma with other laparoscopic methods compared with the nonclosure IPOM technique.

## ACKNOWLEDGMENTS

The authors would like to thank Balla Fathalla, MD, for the schematic illustrations for this study.

## Supplementary Material


