# Supplementary material for: Peritoneal Bridging Versus Nonclosure in Laparoscopic Ventral Hernia Repair: A Randomized Controlled Trial
Source: Ann Surg Open. 2023 Feb 2;4(1):e257. doi: 10.1097/AS9.0000000000000257 (PMC10431530; doi:10.1097/AS9.0000000000000257)
Supplement: Supplementary file 1 [file as9-4-e257-s001.pdf]

Table 1 Temporal evolution in seroma incidence

	Results from present study			Piazzese et al[11]	Susmalian et al[12]
**Postoperative follow-up visits**	sIPOM	IPOM-pb	Total	Non-closure (sIPOM)	Non-closure (sIPOM)
1th month	52/56 (93%)	30/48 (63%)	82/104 (79%)	17/93 (18%)	18/20 (90%)
3rd months	19/52 (37%)	22/49 (45%)	41/101 (41%)	7/93 (7.5%)	4/20 (20%)
6th months	10/39 (26%)	10/31 (32%)	20/70 (29%)	2/93 (2%)	-

Comparison of temporal evolution in seroma incidence between the results by Piazzase et al, Susmalian et al, and present study.
