# Supplementary material for: Peritoneal Bridging Versus Nonclosure in Laparoscopic Ventral Hernia Repair: A Randomized Controlled Trial
Source: Ann Surg Open. 2023 Feb 2;4(1):e257. doi: 10.1097/AS9.0000000000000257 (PMC10431530; doi:10.1097/AS9.0000000000000257)
Supplement: Supplementary file 2 [file as9-4-e257-s002.pdf]

Supplement materials

Table 2. Number of patients who did not attend the follow-up visit (missing data) in each group at the respective follow-up period

**Follow-up (months)**	**sIPOM**		**IPOM-pb**		**Total**	
	Count	Percentage	Count	Percentage	Count	Percentage
1	1	2%	3	6%	4	4%
3	5	9%	2	4%	7	6%
6	18	32%	20	39%	38	35%
12	28	49%	31	61%	59	55%
