# Supplementary material for: Peritoneal Bridging Versus Nonclosure in Laparoscopic Ventral Hernia Repair: A Randomized Controlled Trial
Source: Ann Surg Open. 2023 Feb 2;4(1):e257. doi: 10.1097/AS9.0000000000000257 (PMC10431530; doi:10.1097/AS9.0000000000000257)
Supplement: Supplementary file 3 [file as9-4-e257-s003.pdf]

Supplementary results material

Table 3. Patients who were lost to follow-up and had no seroma in previous visit(s)

**Follow-up (months)**	**sIPOM**		**IPOM-pb**		**Total**	
	Count	Percentage	Count	Percentage	Count	Percentage
1	x	x	x	x	x	x
3	1	20	2	100	3	43
6	14	78	13	65	27	71
12	22	79	21	68	43	73
