# Supplementary material for: Peritoneal Bridging Versus Nonclosure in Laparoscopic Ventral Hernia Repair: A Randomized Controlled Trial
Source: Ann Surg Open. 2023 Feb 2;4(1):e257. doi: 10.1097/AS9.0000000000000257 (PMC10431530; doi:10.1097/AS9.0000000000000257)
Supplement: Supplementary file 4 [file as9-4-e257-s004.pdf]

Supplementary results material

Table 4. Incidence of seroma after including lost of follow-up visits of patients who had no seroma in previous visit(s). χ^2 or *Fisher Exact test was used.

Postoperative follow-up visits	sIPOM (N=60)	IPOM-pb (N=52)	All repairs (N=112)	P value
Seroma (counts)				
1th month	52/56 (93%)	30/48 (63%)	82/104 (79%)	< 0.001
3rd months	19/53 (36%)	22/51 (43%)	41/104 (39%)	0.447
6th months	10/53 (19%)	10/44 (23%)	20/97 (21%)	0.640
12th months	3/51 (6%)	5/41 (12%)	8/92 (9%)	0.459*
